# Impulsivity is the principal correlate of problematic internet use in young adults with attention-deficit/hyperactivity disorder: a cross-sectional clinical study

**DOI:** 10.3389/fpsyt.2026.1974570

**Published:** 2026-10-28

**Authors:** Luca Giacovelli, Davide Fedeli, Vassilis Martiadis, Simone Vanzetto, Matteo Cerioli, Luca Larini, Paola Landi, Viviana Venturi, Vera Viganò, Yacob Levi Reibman, Camilla Gesi, Bernardo Dell’Osso

**Affiliations:** 1 Department of Psychiatry and Addiction, ASST Fatebenefratelli-Sacco, Milan, Italy; 2 Department of Neurology and Neuroscience, Besta, Milan, Italy; 3 Department of Mental Health, ASL Napoli 1 Centro, Naples, Italy; 4 Fondazione Bonolis As.Fra ETS, Vedano al Lambro, Italy; 5 Aldo Ravelli’ Center for Nanotechnology and Neurostimulation, University of Milan, Milan, Italy; 6 Department of Psychiatry and Behavioral Sciences, Stanford University, Stanford, CA, United States

**Keywords:** adults, attention-deficit/hyperactivity disorder, behavioral addiction, dimensional assessment, emotional dysregulation, impulsivity, internet addiction, problematic internet use

## Abstract

**Introduction:**

Attention-deficit/hyperactivity disorder (ADHD) and problematic internet use share clinical features such as impulsivity, emotional dysregulation and impaired inhibitory control, but most evidence comes from children, adolescents and non-clinical samples. It remains unclear which dimension carries this association in adults with a formally established diagnosis.

**Methods:**

Retrospective, cross-sectional study of young adults (aged 18 to 38 years) consecutively assessed at a second-level outpatient service for adult ADHD in Milan, Italy. Diagnosis was established with a semi-structured diagnostic interview (DIVA-5), which also yielded symptom counts for childhood and adult inattention and hyperactivity/impulsivity. Participants completed the Internet Addiction Test, the Barratt Impulsiveness Scale, the Difficulties in Emotion Regulation Scale, measures of depressive and anxiety symptoms, a quality-of-life questionnaire and two ADHD symptom questionnaires. Impulsivity and emotional dysregulation were prespecified variables of interest. Analyses comprised correlations with correction for multiple comparisons, group comparisons, multivariable linear regression and bootstrapped mediation.

**Results:**

Two hundred and fourteen patients had a valid Internet Addiction Test score (mean 52.96, standard deviation 15.16); 22.9% were average users, 62.1% problematic users and 15.0% highly problematic users. Problematic internet use correlated with impulsivity (rho = 0.345) and emotional dysregulation (rho = 0.229), both surviving corrections, while no significant association was detected with the four interview-derived symptom counts (rho from 0.004 to 0.119), the two symptom questionnaires, quality of life or trait anxiety. In the multivariable model impulsivity was the strongest correlate (b = 0.317, p < 0.001; standardized beta 0.33), whereas emotional dysregulation was weaker and varied across specifications (b = 0.091, p = 0.052). Severity, presentation, symptom counts and demographic and clinical covariates were not significantly associated with the outcome. Mediation analysis identified an indirect association between severity and internet use through impulsivity (indirect effect 1.56, 95% confidence interval 0.41 to 2.96).

**Discussion:**

In young adults with ADHD, impulsivity is the dimension most consistently related to problematic internet use, whereas no association was detected with core symptom severity; the confidence intervals remain compatible with weak associations. Assessment of online behavior should not be restricted to the most severely affected patients. The design does not support causal inference.

## 1 Introduction

Attention-deficit/hyperactivity disorder (ADHD) is a childhood-onset, lifespan neurodevelopmental disorder characterized by persistent inattention, hyperactivity and impulsivity (1). Beyond these core features, adults with ADHD commonly report restlessness, sleep disturbance, executive dysfunction and emotional dysregulation (2, 3). These characteristics are associated with impaired occupational and social functioning (4), lower educational attainment (5), poorer general mental health (6) and higher rates of substance use disorders (7).

The estimated prevalence of ADHD is 5 to 8% in children (8) and approximately 2.8% in adults, with a male to female ratio between 1.8:1 and 2.5:1 (5). Although hyperactivity tends to decline with age, a substantial proportion of patients continue to report clinically relevant symptoms in adulthood, most often inattention and emotional dysregulation (3). In psychiatric populations the prevalence is considerably higher, between 15.3% and 19.3% (9). Adult ADHD nonetheless remains frequently underdiagnosed or misdiagnosed in mental health settings because of symptom overlap with bipolar disorder, borderline personality disorder and substance use disorders (10–12). Early and accurate identification matters, since appropriate treatment reduces both ADHD-related impairment and the severity of comorbid conditions (13, 14).

Problematic internet use, often referred to as internet addiction, describes a pattern of excessive preoccupation, urges or compulsive online activity associated with distress or functional impairment (15). It is most prevalent among adolescents and young adults, who are most exposed to gaming and social networking (16, 17), and concern about dysregulated use in vulnerable populations grew during the COVID-19 pandemic (18). Problematic internet use is not a diagnostic category in DSM-5-TR, and the eleventh revision of the International Classification of Diseases recognizes only gaming disorder among online behavioral addictions (1, 19); consensus on definition, thresholds and diagnostic criteria is still lacking (20, 21). Throughout this article the term problematic internet use denotes this dimensional construct; internet addiction is used only when reporting the name of an instrument or the terminology of a cited study, and the term behavioral addiction is used only for the broader diagnostic debate.

Research consistently indicates that problematic internet use and ADHD share features such as impulsivity, poor emotional regulation and executive dysfunction (22, 23). Evidence, however, comes predominantly from pediatric populations and from non-clinical samples, and studies are heterogeneous in the instruments used and in the type of online activity considered (22, 24). In the general adult population, ADHD symptoms assessed by questionnaire are associated with problematic internet use, and this association appears to be partly mediated by impulsivity and by anxious and depressive symptoms (25). In clinically diagnosed adults the evidence is much thinner: a recent case-control study found higher internet use, impulsivity and emotion dysregulation scores in adults with ADHD than in controls, but no significant association between internet use severity and the other measures within the ADHD group (26).

Two questions therefore remain open in clinically diagnosed adults, in whom psychiatric comorbidity and pharmacological treatment are almost universal. The first is whether problematic internet use tracks the severity of the core ADHD symptom dimensions or the associated dimensions of impulsivity and emotional dysregulation. The second is whether the association between overall ADHD severity and problematic internet use, repeatedly reported at the bivariate level, persists after adjustment for those dimensions. We addressed both questions in a clinical sample assessed at a specialist outpatient service, using the symptom counts generated by the diagnostic interview alongside the usual questionnaire measures. We hypothesized that problematic internet use would be associated with impulsivity and emotional dysregulation but not with the severity of core ADHD symptoms.

## 2 Materials and methods

### 2.1 Study design and setting

This was a retrospective, cross-sectional study based on clinical data routinely collected at the Adult ADHD outpatient service of the Department of Mental Health and Addiction, ASST Fatebenefratelli-Sacco, Milan, Italy, a second-level service that provides diagnostic assessment and treatment for adults with suspected or confirmed ADHD and receives referrals from first-level adult and adolescent psychiatric services. The study population comprised all patients whose first access to the service occurred between January 2025 and April 2026. Data were extracted from their clinical records. All measures had been administered as part of routine clinical care, before and independently of the present analysis, and data were anonymized before analysis. Treatment follows international consensus recommendations (2). The study is reported in accordance with the STROBE statement and with the RECORD extension for studies conducted using routinely collected health data (27, 28).

### 2.2 Participants and flow

Inclusion criteria were an age of 18 years or older, a diagnosis of ADHD according to DSM-5 criteria established through comprehensive clinical evaluation, which included a semi-structured diagnostic interview in all but six patients whose diagnosis had been established before referral, and sufficient command of Italian to complete the self-report instruments. Exclusion criteria were an ongoing psychotic episode and severe intellectual disability.

The service database contained 527 records for the study period, of which 251 corresponded to patients with a completed baseline clinical assessment; the remaining records were of patients whose assessment was not completed at the service. A valid total score on the Internet Addiction Test was available for 214 of these patients, who constituted the analytic sample, while 37 had no usable score. Within the analytic sample the diagnostic interview had been administered and its ratings recorded for 208 patients, of whom 206 also had a severity rating; the remaining 6 patients had received their ADHD diagnosis before referral and were in ongoing treatment at the time of assessment, and their interview ratings were not recorded. Patients with and without a usable Internet Addiction Test score did not differ significantly in age (26.1 versus 26.8 years, p = 0.477), sex (p = 0.286) or ADHD severity (p = 0.251), and the distribution of clinical presentation differed at trend level (p = 0.060). The numbers at each stage are shown in **Figure 1** and correspond to the valid n reported for every variable in **Table 1**.

**Figure 1:**
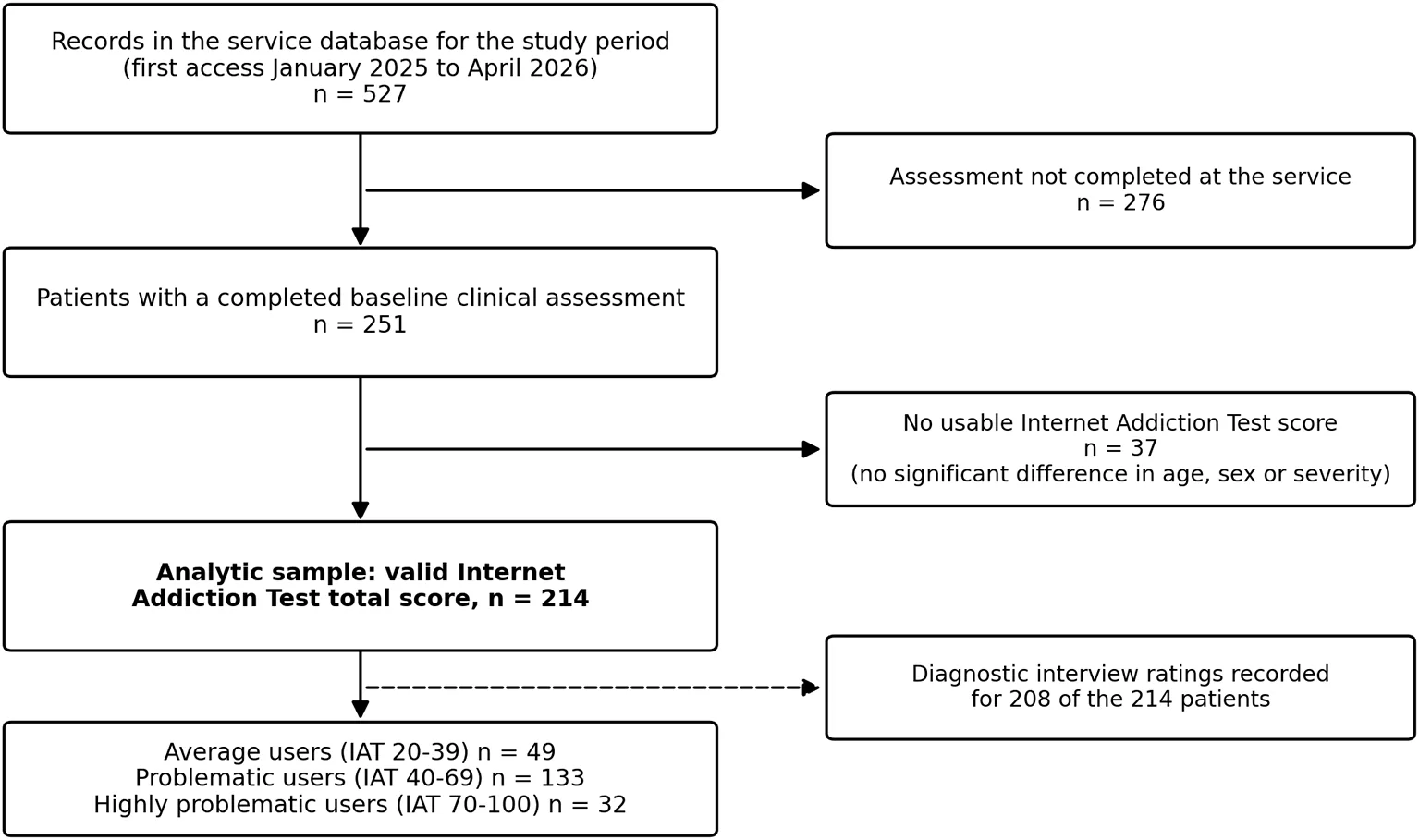
Flow of patients from the service database to the analytic sample. Patients with a completed baseline assessment but no usable Internet Addiction test score did not differ significantly from those included in age, sex or ADHD severity.

**Table 1:** Sociodemographic, clinical and psychometric characteristics of the sample (N = 214).

Characteristic	n (%) or mean (SD)	Valid n
Age, years	26.1 (4.9)	214
Age range, years	18 to 38	214
Female sex	89 (41.8)	213
Age at diagnosis, years	23.5 (6.9)	214
Educational level		
Primary school	1 (0.5)	214
Middle school	35 (16.4)	214
High school	115 (53.7)	214
University degree or higher	63 (29.4)	214
ADHD presentation		
Combined	162 (77.9)	208
Predominantly inattentive	43 (20.7)	208
Predominantly hyperactive/impulsive	3 (1.4)	208
ADHD severity		
Mild	23 (11.2)	206
Moderate	128 (62.1)	206
Severe	55 (26.7)	206
School history		
School difficulties	200 (93.5)	214
Grade retention	137 (64.0)	214
School dropout	78 (36.4)	214
Clinical variables		
Any lifetime psychiatric comorbidity	162 (75.7)	214
Past substance use disorder	75 (35.2)	213
Current substance use disorder	53 (24.9)	213
Sleep disturbance	156 (73.2)	213
Positive MDQ screening	43 (23.9)	180
Lifetime psychiatric hospitalization	29 (13.6)	213
Family history of ADHD	39 (18.3)	213
Family history of psychiatric disorders	119 (55.9)	213
Current ADHD treatment recorded		
Methylphenidate	99 (46.5)	213
Atomoxetine	80 (37.6)	213
Other or unspecified	34 (16.0)	213
Interview-derived symptom counts (0 to 9)		
Adult inattention	7.54 (1.48)	205
Adult hyperactivity/impulsivity	6.48 (2.03)	205
Childhood inattention	7.48 (1.46)	206
Childhood hyperactivity/impulsivity	6.41 (2.39)	206
Concomitant psychotropic treatment		
Antidepressants	91 (42.7)	213
Benzodiazepines	57 (26.8)	213
Mood stabilizers	41 (19.2)	213
Antipsychotics	30 (14.1)	213
Ongoing psychotherapy	90 (42.3)	213
Psychometric measures		
IAT total	52.96 (15.16)	214
BIS-11 total	81.79 (16.05)	211
DERS total	114.11 (24.48)	198
Zung total	47.76 (11.06)	179
STAI-Y1, state anxiety	54.22 (11.72)	180
STAI-Y2, trait anxiety	58.43 (10.26)	181
AAQoL total	43.23 (15.08)	204
ASRS Part A	13.58 (2.94)	204
WURS total	51.98 (15.77)	186
Category of internet use		
Average users (IAT 20 to 39)	49 (22.9)	214
Problematic users (IAT 40 to 69)	133 (62.1)	214
Highly problematic users (IAT 70 to 100)	32 (15.0)	214

Percentages are computed on the number of patients with valid data for each variable, reported in the last column. AAQoL, Adult ADHD Quality of Life questionnaire; ASRS, Adult ADHD Self-Report Scale; BIS-11, Barratt Impulsiveness Scale; DERS, Difficulties in Emotion Regulation Scale; IAT, Internet Addiction Test; MDQ, Mood Disorder Questionnaire; SD, standard deviation; STAI-Y, State-Trait Anxiety Inventory; WURS, Wender Utah Rating Scale.

### 2.3 Measures

ADHD diagnosis, symptom counts, presentation and severity. Diagnosis was established with the Diagnostic Interview for ADHD in Adults, fifth version (DIVA-5), a semi-structured interview based on the DSM-5 criteria for ADHD and one of the instruments recommended by the European consensus statement (1, 2, 29). The DIVA-5 covers the eighteen DSM-5 symptom criteria in childhood and in adulthood, age at onset before the age of 12 years, and impairment in at least two of five life domains (work and education, relationships and family, social contacts, free time and hobbies, self-confidence and self-image). It was administered at the service by psychiatrists and clinical psychologists with specific training and clinical experience in adult ADHD, and collateral information from a parent or partner was sought whenever available, as recommended by the instrument. A diagnosis required at least five of nine symptoms in adulthood and at least six of nine in childhood in the corresponding dimension, together with onset, pervasiveness and impairment; severity was rated by the interviewer on the DSM-5 mild, moderate and severe categories. Inter-rater reliability was not formally assessed at the service and is reported as a limitation. The interview generated the number of criteria met for inattention and for hyperactivity/impulsivity, separately for childhood and adulthood (0 to 9 each), the clinical presentation (predominantly inattentive, predominantly hyperactive/impulsive, combined) and an ordinal severity rating (mild, moderate, severe). The four symptom counts were used as the primary measure of core symptom load, because they derive from the diagnostic interview rather than from screening questionnaires.

ADHD symptom questionnaires. The Wender Utah Rating Scale (WURS) provides a retrospective self-report measure of childhood ADHD symptoms (30), and Part A of the Adult ADHD Self-Report Scale (ASRS-v1.1) is a six-item screener of current symptoms with total scores from 0 to 24 (31). Both were treated as secondary measures of symptom load, since neither is a comprehensive clinical measure of severity.

Impulsivity. The Barratt Impulsiveness Scale, version 11 (BIS-11), is a 30-item self-report questionnaire rated from 1 (rarely or never) to 4 (almost always or always), with some items reverse-scored; total scores range from 30 to 120 and higher scores indicate greater impulsiveness (32). The Italian version was used (33).

Emotional dysregulation. The Difficulties in Emotion Regulation Scale (DERS) is a 36-item self-report measure with six subscales; total scores range from 36 to 180, higher scores indicating greater dysregulation (34). The Italian adaptation of the full 36-item version was used (35).

Depressive and anxiety symptoms. The Zung Self-Rating Depression Scale is a 20-item measure of depressive symptoms with raw total scores ranging from 20 to 80 (36, 37), validated in Italian (38). The State-Trait Anxiety Inventory, form Y (STAI-Y), comprises two 20-item subscales assessing current (state, STAI-Y1) and habitual (trait, STAI-Y2) anxiety, each ranging from 20 to 80 (39); the Italian version was used (40). The Mood Disorder Questionnaire (MDQ) was used to screen for bipolar spectrum disorders (41), in its Italian version (42).

Quality of life. The Adult ADHD Quality of Life questionnaire (AAQoL) is a 29-item disease-specific measure of health-related quality of life over the preceding two weeks, yielding a total score from 0 to 100, with higher scores indicating better quality of life (43, 44).

Problematic internet use. The Internet Addiction Test (IAT) is a 20-item self-report questionnaire covering the impact of internet use on daily routine, social life, productivity, sleep and mood, with total scores ranging from 20 to 100 (45). Its psychometric properties have been examined extensively (46, 47), including in Italian (48). Following the original scoring convention, scores of 20 to 39 identify average users with full control over their use, scores of 40 to 69 identify frequent problems related to internet use, and scores of 70 to 100 indicate that internet use is causing significant problems. The total score was the dependent variable in all primary analyses; the three-level classification was used only for descriptive group comparisons, and no analysis was performed at the level of the IAT subscales.

Clinical and sociodemographic variables. Age, sex, educational level, age at diagnosis, history of grade retention and school dropout, lifetime psychiatric comorbidity, past and current substance use disorder, sleep disturbance, family history of ADHD and of psychiatric disorders, lifetime psychiatric hospitalization, ongoing ADHD treatment, concomitant psychotropic treatment and ongoing psychotherapy were retrieved from the clinical records.

### 2.4 Statistical analysis

Impulsivity and emotional dysregulation were prespecified as the variables of primary interest; all other comparisons are exploratory and are identified as such. Descriptive statistics are reported as mean and standard deviation for continuous variables and as absolute frequency and percentage of valid observations for categorical variables. No transformation was applied to the psychometric scores. Analyses were performed on complete cases and the number of observations available is reported for every analysis; missing data ranged from 1.4% for the BIS-11 to 16.4% for the Zung scale.

The distribution of continuous variables was examined with the Shapiro-Wilk test; because most measures deviated from normality, bivariate associations were tested with Spearman rank correlations. All 36 pairwise correlations among the nine psychometric measures were computed simultaneously and p values were adjusted with the Holm-Bonferroni procedure (49). The four interview-derived symptom counts were correlated with the IAT total score as a separate, prespecified family of four tests, also adjusted with the Holm-Bonferroni procedure.

Patients were classified according to the IAT total score as average users (20 to 39), problematic users (40 to 69) or highly problematic users (70 to 100). Continuous variables were compared across the three groups with the Kruskal-Wallis test and categorical variables with the chi-square test; because these comparisons involve eighteen omnibus tests, p values were adjusted with the Benjamini-Hochberg procedure and both unadjusted and adjusted values are reported. Where the omnibus test remained significant after adjustment, Dunn *post hoc* comparisons with Holm correction were performed. Comparisons of the IAT total score between two independent groups defined by clinical variables were performed with the Mann-Whitney U test and were likewise adjusted with the Benjamini-Hochberg procedure across that family of tests.

A multivariable linear regression was fitted with the IAT total score as the dependent variable. Predictors were chosen *a priori* on clinical grounds and comprised sex, age, educational level, ADHD severity, clinical presentation, current substance use disorder, any lifetime psychiatric comorbidity, DERS total score and BIS-11 total score. Clinical presentation was coded as combined versus non-combined, because only three patients had a predominantly hyperactive/impulsive presentation; ADHD severity and educational level were entered as ordinal variables. Normality and homoscedasticity of residuals were verified with the Shapiro-Wilk and Breusch-Pagan tests, independence with the Durbin-Watson statistic, and collinearity with the variance inflation factor. Standardized coefficients and the increment in explained variance attributable to each variable of interest are reported alongside unstandardized coefficients. Prespecified sensitivity analyses used heteroscedasticity-consistent (HC3) standard errors and added, in turn, the interview-derived symptom counts, the two symptom questionnaires, ongoing psychotropic treatment, depressive and state anxiety symptoms, and categorical rather than ordinal coding of severity and education. A multiple-imputation analysis and an ordinal logistic model on the three IAT categories were performed as further sensitivity analyses. All sensitivity analyses are reported in **Supplementary Table 1**.

A parallel mediation model was estimated with ADHD severity as the independent variable, BIS-11 and DERS total scores as parallel mediators, IAT total score as the dependent variable, and sex, age and educational level as covariates. Indirect effects were estimated with 5,000 bootstrap resamples and 95% percentile confidence intervals (50). Because all variables were measured at the same time point, the model decomposes an association and does not establish temporal or causal ordering. Statistical significance was set at a two-tailed p of 0.05. Analyses were performed in Python 3 using the pandas, SciPy, statsmodels and scikit-posthocs libraries, which were used for the bootstrapped mediation, the heteroscedasticity-consistent estimator, the multiple imputation and the Dunn *post hoc* comparisons.

### 2.5 Ethics statement

The study was conducted in accordance with the Declaration of Helsinki. All patients had signed, at the time of their first access to the service, the informed consent forms used in routine care, covering treatment, the psychodiagnostic procedures and the use of their data in aggregated and anonymized form for teaching and research purposes. Because the study consisted of a retrospective analysis of anonymized data collected during routine clinical care, and was covered by that consent, further ethics committee approval was not required. No identifying information is reported.

## 3 Results

### 3.1 Sample characteristics

The analytic sample comprised 214 patients (**Figure 1**). Their sociodemographic and clinical characteristics are reported in **Table 1**. Mean age was 26.1 years (standard deviation, SD, 4.9; range 18 to 38) and 89 patients (41.8%) were women. Mean age at diagnosis was 23.5 years (SD 6.9). The combined presentation was the most frequent (162 of 208 with a valid rating, 77.9%), followed by the predominantly inattentive (43, 20.7%) and the predominantly hyperactive/impulsive presentation (3, 1.4%); ADHD was rated moderate in 128 patients (62.1%), severe in 55 (26.7%) and mild in 23 (11.2%). Mean interview-derived symptom counts were 7.5 (SD 1.5) for adult inattention, 6.5 (SD 2.0) for adult hyperactivity/impulsivity, 7.5 (SD 1.5) for childhood inattention and 6.4 (SD 2.4) for childhood hyperactivity/impulsivity.

School difficulties were reported by 200 patients (93.5%), grade retention by 137 (64.0%) and school dropout by 78 (36.4%). A lifetime psychiatric comorbidity was present in 162 patients (75.7%), a past substance use disorder in 75 (35.2%) and a current substance use disorder in 53 (24.9%); 156 patients (73.2%) reported sleep disturbance and 29 (13.6%) had a history of psychiatric hospitalization. At assessment, 99 patients (46.5%) were receiving methylphenidate and 80 (37.6%) atomoxetine, while for 34 (16.0%) the recorded ADHD treatment was of another or unspecified type. Concomitant psychotropic treatment included antidepressants in 91 patients (42.7%), benzodiazepines in 57 (26.8%), mood stabilizers in 41 (19.2%) and antipsychotics in 30 (14.1%); 90 patients (42.3%) were in psychotherapy.

The mean IAT total score was 52.96 (SD 15.16; range 20 to 91; median 54). According to the standard cut-offs, 49 patients (22.9%) were classified as average users, 133 (62.1%) as problematic users and 32 (15.0%) as highly problematic users. Mean scores on the other instruments were 81.79 (SD 16.05) for the BIS-11, 114.11 (SD 24.48) for the DERS, 47.76 (SD 11.06) for the Zung scale, 54.22 (SD 11.72) for state anxiety, 58.43 (SD 10.26) for trait anxiety, 43.23 (SD 15.08) for the AAQoL, 13.58 (SD 2.94) for the ASRS and 51.98 (SD 15.77) for the WURS. Forty-three of the 180 patients with a valid MDQ (23.9%) screened positive for the bipolar spectrum.

### 3.2 Problematic internet use and clinical dimensions

Spearman correlations among the nine psychometric measures are reported in **Table 2**. The IAT total score correlated positively with impulsivity (rho = 0.345, n = 211, p < 0.001) and with emotional dysregulation (rho = 0.229, n = 198, p = 0.001); both associations survived Holm-Bonferroni correction across the 36 pairwise tests (adjusted p < 0.001 and p = 0.022). The correlations with depressive symptoms (rho = 0.205, n = 179) and state anxiety (rho = 0.205, n = 180) were significant before but not after correction (unadjusted p = 0.006 for both, adjusted p = 0.106), and no association emerged with trait anxiety (rho = 0.143, p = 0.054) or quality of life (rho = -0.082, p = 0.245).

**Table 2:** Spearman rank correlations among the psychometric measures.

Measure	Zung	STAI-Y1	STAI-Y2	DERS	BIS-11	IAT	AAQoL	ASRS	WURS
Zung	1								
STAI-Y1	0.615***	1							
STAI-Y2	0.569***	0.728***	1						
DERS	0.368***	0.385***	0.304***	1					
BIS-11	0.110	0.186	0.067	0.256**	1				
IAT	0.205	0.205	0.143	0.229*	0.345***	1			
AAQoL	-0.258*	-0.309***	-0.271**	-0.336***	-0.188	-0.082	1		
ASRS	0.161	0.067	0.028	0.279**	0.169	0.082	-0.121	1	
WURS	0.285**	0.328***	0.146	0.369***	0.248*	0.090	-0.057	0.110	1

Values are Spearman rho coefficients. Pairwise complete observations, n from 172 to 211. Asterisks refer to p values adjusted with the Holm-Bonferroni procedure across all 36 pairwise tests: *p < 0.05, **p < 0.01, ***p < 0.001. Abbreviations as in **Table 1**.

No significant association was detected between the IAT total score and any of the four interview-derived symptom counts: adult inattention rho = 0.012 (n = 205, 95% confidence interval -0.125 to 0.149, p = 0.866), adult hyperactivity/impulsivity rho = 0.119 (n = 205, -0.018 to 0.252, p = 0.088), childhood inattention rho = 0.004 (n = 206, -0.132 to 0.141, p = 0.951) and childhood hyperactivity/impulsivity rho = 0.080 (n = 206, -0.058 to 0.214, p = 0.255); none approached significance after Holm-Bonferroni correction within this family of four tests (**Supplementary Table 3**). The confidence intervals exclude associations stronger than about 0.25 but are compatible with weak associations, so these results indicate that no association was detected and not that none exists. The same held for the two symptom questionnaires (ASRS rho = 0.082, n = 204, 95% confidence interval -0.056 to 0.217, p = 0.243; WURS rho = 0.090, n = 186, -0.055 to 0.231, p = 0.223), and for quality of life (rho = -0.082, -0.217 to 0.056) and trait anxiety (rho = 0.143, -0.003 to 0.283). These measures were not inert in the present sample: the adult and childhood hyperactivity/impulsivity counts correlated with emotional dysregulation (rho = 0.224 and 0.218, p = 0.002 for both), and the WURS correlated with emotional dysregulation (rho = 0.369) and with impulsivity (rho = 0.248), both surviving correction. Impulsivity as measured by the BIS-11 was only weakly related to the interview-derived hyperactivity/impulsivity counts, in adulthood (rho = 0.088, n = 202, p = 0.211) and in childhood (rho = 0.134, n = 203, p = 0.057), and to the ASRS (rho = 0.169, n = 201, p = 0.016), while it correlated more strongly with the WURS (rho = 0.248, n = 183, p = 0.001) and with the DERS (rho = 0.256, n = 195, p < 0.001).

Among the remaining associations, emotional dysregulation correlated with depressive symptoms (rho = 0.368), state anxiety (rho = 0.385), trait anxiety (rho = 0.304) and impulsivity (rho = 0.256), all surviving correction, and quality of life correlated inversely with depressive symptoms (rho = -0.258), state anxiety (rho = -0.309), trait anxiety (rho = -0.271) and emotional dysregulation (rho = -0.336).

### 3.3 Comparisons across categories of internet use

Comparisons among average, problematic and highly problematic users are reported in **Table 3**. After adjustment for the eighteen omnibus tests, only the two prespecified measures remained significant: impulsivity (H = 15.67, unadjusted p < 0.001, adjusted p = 0.005) and emotional dysregulation (H = 15.15, unadjusted p < 0.001, adjusted p = 0.005). *Post hoc* comparisons showed that impulsivity separated all three groups (average versus problematic users p = 0.028, problematic versus highly problematic users p = 0.014, average versus highly problematic users p < 0.001), whereas emotional dysregulation separated average users from the other two groups (p = 0.004 and p < 0.001) without distinguishing problematic from highly problematic users (p = 0.115). State anxiety, current ADHD symptom load on the ASRS, current substance use disorder, sleep disturbance and sex distribution differed at unadjusted p < 0.05 but not after adjustment (adjusted p from 0.091 to 0.129); age, age at diagnosis, depressive symptoms, trait anxiety, quality of life, the WURS, past substance use disorder, psychiatric comorbidity, hospitalization, MDQ screening status and antidepressant treatment did not differ.

**Table 3:** Comparison of clinical measures across categories of internet use.

Measure	Average users	Problematic users	Highly problematic users	H or chi-square	p	p (BH)	*Post hoc* (Dunn, Holm)
BIS-11 total	75.43 (12.77) [49]	82.18 (16.27) [131]	90.19 (15.96) [31]	15.67	<0.001	0.005	AU vs PU p = 0.028; PU vs HPU p = 0.014; AU vs HPU p < 0.001
DERS total	101.65 (27.81) [46]	116.55 (22.40) [123]	123.48 (20.42) [29]	15.15	<0.001	0.005	AU vs PU p = 0.004; AU vs HPU p < 0.001
Zung total	46.03 (11.24) [38]	47.76 (10.91) [114]	50.19 (11.45) [27]	2.55	0.280	0.388	—
STAI-Y1	50.95 (12.27) [38]	54.32 (11.43) [115]	58.41 (11.16) [27]	7.02	0.030	0.091	—
STAI-Y2	56.11 (11.29) [38]	58.53 (10.13) [116]	61.30 (8.80) [27]	3.21	0.201	0.329	—
AAQoL total	44.92 (13.99) [45]	43.58 (14.77) [131]	38.86 (17.74) [28]	1.82	0.402	0.482	—
ASRS Part A	13.23 (2.91) [47]	13.41 (3.05) [126]	14.77 (2.17) [31]	7.07	0.029	0.091	—
WURS total	50.88 (19.62) [41]	52.50 (14.13) [116]	51.48 (16.44) [29]	0.29	0.864	0.864	—
Age, years	25.71 (4.39) [49]	25.93 (5.06) [133]	27.25 (4.99) [32]	2.31	0.314	0.404	—
Age at diagnosis, years	22.98 (6.32) [49]	23.20 (7.33) [133]	25.84 (5.61) [32]	4.23	0.120	0.269	—
Categorical variables, % positive							
Female sex	55.1	35.6	46.9	5.99	0.050	0.129	—
Current substance use disorder	24.5	20.5	43.8	7.48	0.024	0.091	—
Past substance use disorder	32.7	32.6	50.0	3.61	0.164	0.296	—
Psychiatric comorbidity	79.6	72.9	81.2	1.49	0.474	0.533	—
Sleep disturbance	61.2	74.2	87.5	7.00	0.030	0.091	—
Lifetime hospitalization	12.2	12.9	18.8	0.86	0.652	0.690	—
Positive MDQ screening	28.9	19.3	35.7	4.01	0.135	0.269	—
Antidepressant treatment	32.7	46.2	43.8	2.70	0.259	0.388	—

Continuous variables are mean (SD) [n] and were compared with the Kruskal-Wallis test, followed by Dunn *post hoc* comparisons with Holm correction when the omnibus test was significant. Categorical variables are percentages of patients with valid data and were compared with the chi-square test (2 degrees of freedom). p (BH) is the p value adjusted with the Benjamini-Hochberg procedure across the eighteen omnibus tests reported in this table; Dunn *post hoc* comparisons were performed only where the adjusted omnibus p value remained below 0.05. Average users, IAT 20 to 39; problematic users, IAT 40 to 69; highly problematic users, IAT 70 to 100. Abbreviations as in **Table 1**.

In exploratory comparisons of the IAT total score between groups defined by clinical variables, unadjusted differences emerged for grade retention (55.08 versus 49.18, p = 0.005), sleep disturbance (54.51 versus 48.63, p = 0.014), antidepressant treatment (55.08 versus 51.42, p = 0.041) and ADHD severity (49.00, 51.87 and 57.60 from mild to severe, p = 0.037), but none survived Benjamini-Hochberg adjustment across this family of fourteen tests (adjusted p from 0.068 to 0.951). The IAT total score did not differ by sex, clinical presentation, educational level, MDQ status, psychiatric comorbidity or history of hospitalization.

### 3.4 Multivariable model

The multivariable linear regression is reported in **Table 4** and the standardized coefficients in **Figure 2**. The model was significant (F(9,181) = 4.27, p < 0.001) and explained 17.5% of the variance in the IAT total score (adjusted R-squared 0.134, n = 191). Impulsivity was the strongest correlate (b = 0.317, standard error 0.071, t = 4.49, p < 0.001, 95% confidence interval 0.178 to 0.457; standardized beta 0.33), corresponding to an increase of about 3.2 IAT points for every 10 additional BIS-11 points and to an increment in explained variance of 0.092. Emotional dysregulation was weaker and did not reach the conventional threshold (b = 0.091, p = 0.052; standardized beta 0.15; increment in explained variance 0.018). ADHD severity was not significantly associated with the outcome (b = 2.41, 95% confidence interval -1.39 to 6.21, p = 0.212; standardized beta 0.10), and neither were sex, age, education, clinical presentation, current substance use disorder or psychiatric comorbidity; the confidence intervals for these predictors are wide and do not exclude associations of small magnitude. Residuals were normally distributed (Shapiro-Wilk p = 0.369) and homoscedastic (Breusch-Pagan p = 0.399), the Durbin-Watson statistic was 1.86 and the maximum variance inflation factor was 1.31.

**Table 4:** Multivariable linear regression with the IAT total score as the dependent variable.

Predictor	b	SE	t	p	95% CI
Intercept	11.704	9.799	1.19	0.234	-7.631 to 31.040
Female sex	-1.420	2.177	-0.65	0.515	-5.716 to 2.877
Age	0.163	0.232	0.70	0.483	-0.295 to 0.620
Educational level	-0.455	1.739	-0.26	0.794	-3.886 to 2.977
ADHD severity	2.412	1.925	1.25	0.212	-1.386 to 6.211
Combined vs non-combined presentation	-0.913	2.694	-0.34	0.735	-6.228 to 4.402
Current substance use disorder	0.121	2.705	0.04	0.964	-5.217 to 5.458
Any psychiatric comorbidity	-2.609	2.573	-1.01	0.312	-7.686 to 2.468
DERS total	0.091	0.046	1.96	0.052	-0.001 to 0.183
BIS-11 total	0.317	0.071	4.49	<0.001	0.178 to 0.457

n = 191; F(9, 181) = 4.27, p < 0.001; R-squared = 0.175, adjusted R-squared = 0.134. Clinical presentation was coded as combined versus non-combined; reference categories were male sex, non-combined presentation, absence of current substance use disorder and absence of psychiatric comorbidity. ADHD severity and educational level were entered as ordinal variables. Standardized coefficients were 0.33 for the BIS-11, 0.15 for the DERS and 0.10 for ADHD severity, with increments in explained variance of 0.092, 0.018 and 0.007 respectively. Maximum variance inflation factor 1.31; Shapiro-Wilk test on residuals p = 0.369; Breusch-Pagan test p = 0.399. Sensitivity analyses are reported in **Supplementary Table 1**. CI, confidence interval; SE, standard error; other abbreviations as in **Table 1**.

**Figure 2:**
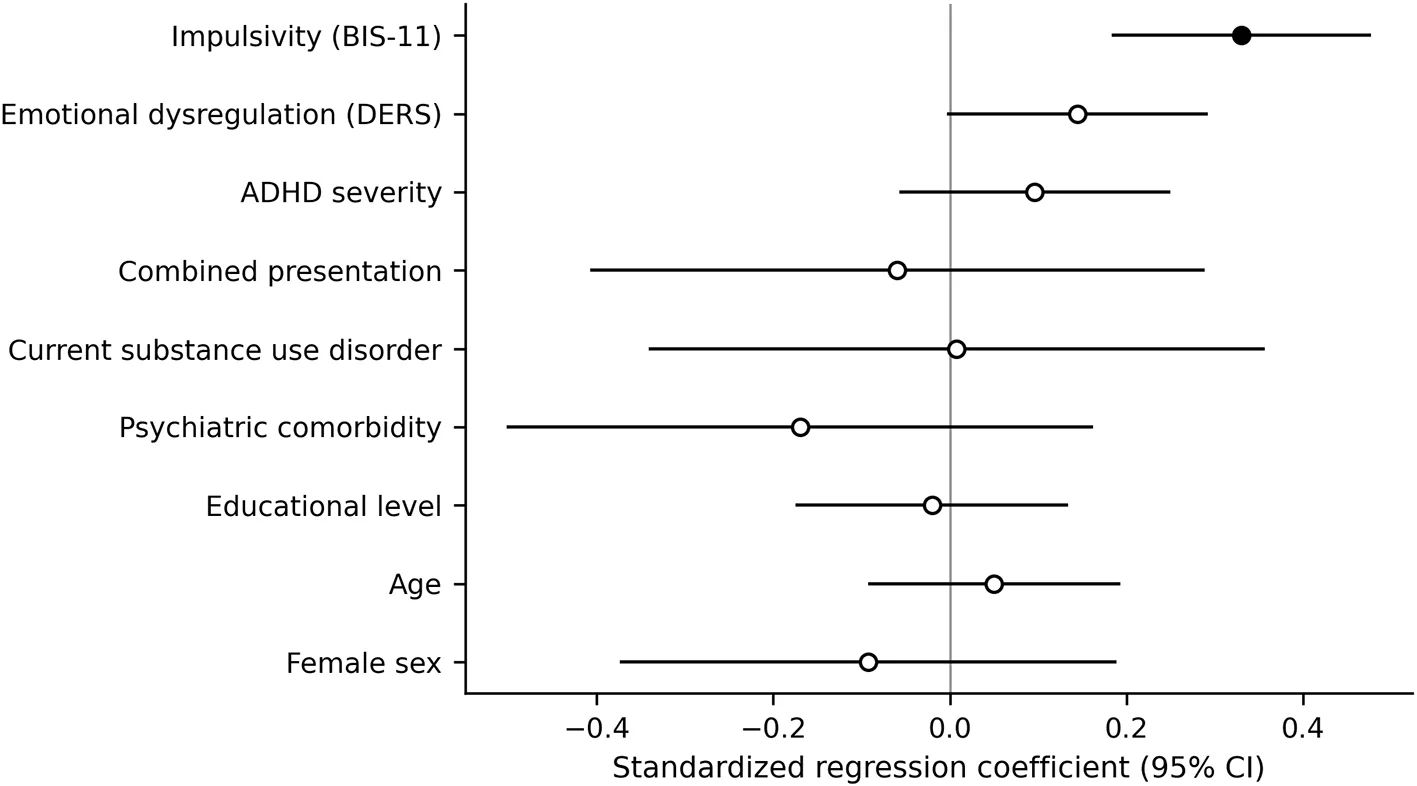
Standardized regression coefficients with 95% confidence intervals for the multivariable model of the internet addiction test total score (n = 191). Filled markers indicate coefficients significant at p < 0.05. BIS-11, Barratt Impulsiveness Scale; DERS, Difficulties in Emotion Regulation Scale.

Sensitivity analyses are reported in **Supplementary Table 1**. The coefficient for impulsivity was essentially unchanged with HC3 standard errors (b = 0.317, p < 0.001), when the interview-derived symptom counts were added (b = 0.326, p < 0.001, with adult inattention p = 0.260 and adult hyperactivity/impulsivity p = 0.378), when the two symptom questionnaires were added (b = 0.318, p < 0.001, with ASRS p = 0.587 and WURS p = 0.164), when ongoing psychotropic treatment was added (b = 0.304, p < 0.001) and when depressive and state anxiety symptoms were added (p < 0.001). The coefficient for emotional dysregulation varied across specifications: it reached significance when the symptom questionnaires were included (b = 0.113, p = 0.030) and in the multiple-imputation analysis (b = 0.095, p = 0.029), remained borderline with HC3 standard errors (p = 0.066), with the symptom counts (p = 0.060), with psychotropic treatment (p = 0.089) and with categorical coding of severity and education (p = 0.073), and was non-significant when depressive and state anxiety symptoms were included (p = 0.109). In an ordinal logistic model on the three categories of internet use, both impulsivity (odds ratio per 10 points 1.37, p = 0.001) and emotional dysregulation (odds ratio per 10 points 1.25, p < 0.001) were associated with the outcome.

The 23 patients excluded from the main model because of missing covariates (**Supplementary Table 2**) were younger than those included (24.5 versus 26.3 years, p = 0.041) but did not differ in IAT total score (p = 0.319), impulsivity (p = 0.114), emotional dysregulation (p = 0.589), severity (p = 0.768) or sex (p = 0.319). A multiple-imputation analysis including all 214 patients confirmed the coefficient for impulsivity (b = 0.306, p < 0.001) and yielded a significant coefficient for emotional dysregulation (b = 0.095, p = 0.029).

### 3.5 Mediation analysis

In the parallel mediation model (n = 191), ADHD severity predicted both impulsivity (b = 5.22, p = 0.007) and emotional dysregulation (b = 9.80, p < 0.001). With both mediators in the model, impulsivity was associated with the IAT total score (b = 0.299, p < 0.001) whereas emotional dysregulation was not (b = 0.083, p = 0.064). The total effect of ADHD severity on the IAT total score was 4.72 (p = 0.011) and the direct effect was 2.34 (p = 0.194). The indirect effect through impulsivity was statistically detectable (1.56, 95% confidence interval 0.41 to 2.96), while the indirect effect through emotional dysregulation was not (0.81, 95% confidence interval -0.13 to 1.95). The direct effect was therefore estimated imprecisely and was not distinguishable from zero, but it corresponded to about half of the total effect and cannot be regarded as absent.

## 4 Discussion

This study examined which clinical dimension carries the association with problematic internet use in a clinical sample of adults with ADHD. First, problematic internet use was associated with impulsivity and, more weakly, with emotional dysregulation, while no significant association was detected with any measure of core symptom load, whether derived from the diagnostic interview or from questionnaires. Second, impulsivity was the strongest correlate in the multivariable model and the only measure that separated all three categories of internet use, and its coefficient was stable across every sensitivity analysis. Third, the bivariate association between overall ADHD severity and problematic internet use did not persist after adjustment, and the mediation model identified a detectable indirect association through impulsivity. To our knowledge this is among the largest studies to examine problematic internet use in a clinically diagnosed adult ADHD cohort and to compare interview-derived symptom dimensions with impulsivity and emotional dysregulation within the same multivariable framework.

Fewer than one in four patients fell in the range of controlled use, and more than three quarters scored above the threshold indicating frequent or significant problems. Even allowing for the fact that IAT cut-offs were derived in the general population and tend to overestimate clinically meaningful impairment (21, 51), such a distribution indicates that problematic internet use is close to normative in young adults referred to a specialist ADHD service, and that assessing it only when a patient raises the issue will miss most cases.

The central role of impulsivity is consistent with previous work in young adults with probable ADHD (52), in patients with internet gaming disorder (53) and in university students, where impulsivity and inattention tracked the severity of problematic use (54). It also parallels the finding, in a large general-population adult sample, that the association between self-reported ADHD symptoms and problematic internet use is partly mediated by impulsivity (25). Impulsivity is a well-established transdiagnostic risk factor for addictive behavior (55) and a core feature of ADHD, which is itself a recognized predisposing condition for both substance and behavioral addictions (56, 57). Neurobiological work indicates overlapping reward and inhibitory control circuits in problematic online behavior and substance addiction (58, 59), and prefrontal dysfunction during inhibitory tasks has been described in children with ADHD (60); whether the same holds in adults was not tested here. Online environments provide immediate, variable and highly salient reinforcement, the contingency to which a reward system biased toward immediate gratification is most sensitive (61).

The present data suggest that the association is dimension-specific and not severity-related, with the caveat that the absence of a detected association is not evidence of no association. This is the point on which the study adds most, because the symptom counts generated by the diagnostic interview, and not only screening questionnaires, were available: neither adult nor childhood inattention or hyperactivity/impulsivity was associated with problematic internet use, while the same counts did correlate with emotional dysregulation, which argues against a simple lack of sensitivity. The confidence intervals around the symptom-count estimates exclude associations stronger than about 0.25 but remain compatible with weak ones, so the appropriate conclusion is that symptom load is at most weakly related to problematic internet use in this population. Categorical severity ratings compress heterogeneous symptom profiles into a single ordinal variable and appear to be poor proxies for the mechanism at work. In practical terms, an adult with mild ADHD and high impulsivity may show more problematic use than an adult with severe ADHD and low impulsivity, so a severity-based triage would misclassify both. Impulsivity as measured by the BIS-11 and the hyperactivity/impulsivity symptom dimension of the diagnostic interview are conceptually adjacent, but they were only weakly related in this sample (rho = 0.088 in adulthood and 0.134 in childhood), which suggests that the BIS-11 captures a trait disposition towards rapid and unplanned action rather than the symptom criteria used for diagnosis, and makes construct overlap an unlikely explanation of the present findings.

Our findings differ in part from those of a community study in which self-reported inattention was the symptom dimension most strongly related to problematic internet use, with a moderating role of inhibitory control (24). Two differences may account for this. Our patients all met diagnostic criteria, so the range of core symptoms is restricted by design, whereas in community samples symptom scores vary across the whole spectrum. In addition, we had no objective measure of inhibitory control, which may capture a facet of impulsivity that self-report does not. Conversely, the case-control study that reported no within-group associations in adults with ADHD (26) was based on a considerably smaller clinical sample, which limited the power of within-group analyses.

Emotional dysregulation occupies an intermediate position. It correlated with problematic internet use and survived correction, it separated average users from the other two groups, and it was associated with the outcome in the ordinal model, which is coherent with evidence linking problematic internet use to emotion regulation deficits (62) and with work on emotional and metacognitive predictors of internet gaming disorder (63, 64). In the main multivariable model, however, its coefficient fell just short of significance and its magnitude was less than half that of impulsivity, and its significance varied with the specification. Emotional dysregulation affects a large proportion of adults with ADHD and is linked to poorer psychosocial outcomes (65–68); on the present evidence it is best described as a weaker and model-dependent correlate of problematic internet use, and further work with larger samples is needed to establish whether its contribution is independent of impulsivity.

The correlations of problematic internet use with state anxiety and with depressive symptoms were significant before but not after correction for multiple comparisons, and no association emerged with trait anxiety, in partial contrast with previous reports (69–71). That the weak associations observed concerned state and not trait anxiety is compatible with the interpretation that problematic use accompanies current distress and not a stable anxious disposition; this reading remains tentative, since the design does not allow the direction of the relationship to be determined and the high proportion of patients receiving antidepressants at assessment may have attenuated depressive symptom scores. Quality of life was unrelated to problematic internet use even though it was clearly related to depression, anxiety and emotional dysregulation, which suggests that, at these levels of severity, problematic internet use is not yet perceived by patients as a source of functional impairment.

Sleep disturbance, grade retention, antidepressant treatment and ADHD severity were associated with higher IAT scores in unadjusted comparisons, in line with the literature relating problematic internet use to insomnia and to poorer academic outcomes (70, 72, 73). None of these differences survived adjustment for multiple comparisons, however, and none was confirmed in the multivariable model; sleep disturbance in particular is highly prevalent among psychiatric outpatients in Italian community services irrespective of online behavior (74). They are reported for completeness and should be treated as hypothesis-generating.

The construct measured here also needs qualification. Problematic internet use is not a diagnostic entity in DSM-5-TR, and ICD-11 recognizes only gaming disorder among online behavioral addictions (1, 19). The IAT was developed before the current digital environment, its thresholds are conventional and not diagnostic, and its factor structure remains debated (20, 46, 48); for this reason we used the total score as a dimensional measure and did not analyze its subscales. The type of online activity was not recorded, so gaming, social networking, shopping and generalized use cannot be distinguished, and this limits comparison with studies focused on specific online behaviors.

### 4.1 Limitations

Several limitations should be considered. The design is retrospective and cross-sectional and precludes any causal inference; the mediation model decomposes an association measured at a single time point, and reverse or bidirectional relationships between impulsivity and problematic internet use remain entirely plausible. Patients were recruited at a single second-level service and are likely to present with greater severity and higher comorbidity than adults with ADHD in the community, which limits generalizability, and the age range restricts the findings to young adults. Because all patients met diagnostic criteria, the range of core symptom scores is restricted, which may attenuate their association with the outcome. Data were collected during routine care and were not complete for every instrument; complete-case estimates may be biased if missingness was related to unobserved values, and although included and excluded patients differed only in age and a multiple-imputation analysis did not change the main result, residual bias cannot be excluded. All measures other than the diagnostic interview were self-reported and therefore subject to recall and social desirability bias, and no objective index of time spent online or of inhibitory control was available. Because the BIS-11 and the IAT are both self-report instruments, part of their association may reflect shared method variance, whereas the symptom counts derive from an interview; this asymmetry may have favoured the self-report measures, although the weak correlations between the BIS-11 and the interview-derived symptom counts indicate that the two sets of measures capture distinct constructs rather than the same one. The type of online activity was not recorded, so gaming, social networking, shopping and generalized use cannot be distinguished, and the findings refer to problematic internet use as a whole. Psychiatric comorbidity and psychotropic treatment were highly prevalent and were only partially accounted for; treatment was recorded as coded in the clinical charts, and for 16.0% of patients the ADHD treatment recorded could not be attributed to a specific drug class, so treatment-related analyses should be read with caution. ADHD severity was a clinician-assigned ordinal rating whose metric properties are not established, and the thresholds used to classify internet use are conventional and were derived in the general population, so the three categories should not be read as diagnostic groups. The absence of a comparison group without ADHD prevents any conclusion about the specificity of the observed associations. Recruitment covered a single and recent sixteen-month window, so the findings describe the current digital environment and may not generalize to other periods. The sample comprised young adults aged 18 to 38 years, with a mean age of 26 years, so the findings should not be extended to middle-aged or older adults with ADHD. Finally, apart from the two prespecified measures, the analyses are exploratory: adjustment for multiple comparisons was applied within each family of tests, but the number of tests performed means that the weaker findings require replication. The study was not designed as an equivalence study, and the non-significant results reported here should be read as absence of a detected association at the available sample size rather than as evidence of no association.

### 4.2 Clinical implications and future directions

The clinical reading of these findings is that assessment of online behavior in adult ADHD services should not be reserved for patients rated as more severely affected, and that particular attention is warranted when impulsivity is elevated. We did not evaluate the accuracy, sensitivity, specificity or predictive value of any impulsivity threshold, so these data do not support a screening rule based on the BIS-11, and a strategy triggered by impulsivity alone would miss patients with high IAT scores and low impulsivity. Prospective studies are needed to establish whether impulsivity precedes the escalation of problematic use and whether pharmacological or psychological treatment of ADHD modifies its course; a longitudinal follow-up of the present cohort is planned with this aim.

## 5 Conclusion

In young adults with ADHD, impulsivity was the clinical dimension most strongly and most consistently associated with the severity of problematic internet use, whereas no association was detected with core symptom load, measured both by semi-structured interview and by questionnaire, or with overall ADHD severity once impulsivity was taken into account. Emotional dysregulation was a weaker and model-dependent correlate. These findings support a dimensional description of these patients and indicate that problematic internet use should be assessed across the whole severity spectrum, while the confidence intervals around the non-significant estimates leave open the possibility of weak associations with symptom load that a larger sample might detect.

## Data Availability

The raw data supporting the conclusions of this article will be made available by the authors, without undue reservation.
