# Supplementary material for: Impulsivity is the principal correlate of problematic internet use in young adults with attention-deficit/hyperactivity disorder: a cross-sectional clinical study
Source: Front Psychiatry. 2026 Oct 28;17:1974570. doi: 10.3389/fpsyt.2026.1974570 (PMC13752202; doi:10.3389/fpsyt.2026.1974570)
Supplement: Supplementary file 1 [file Table1.docx]

**STROBE and RECORD reporting checklists**

References are given by manuscript section, so that they remain valid through revision.

**STROBE Statement: checklist of items that should be included in reports of cross-sectional studies**

| **Section** | **Item** | **Recommendation** | **Reported in** |
| --- | --- | --- | --- |
| Title and abstract | 1a | Indicate the study's design with a commonly used term in the title or the abstract | Abstract ('Retrospective, cross-sectional study'); title indicates a cross-sectional clinical study |
|  | 1b | Provide in the abstract an informative and balanced summary of what was done and what was found | Abstract |
| Introduction | 2 | Explain the scientific background and rationale for the investigation being reported | Introduction, first four paragraphs |
|  | 3 | State specific objectives, including any prespecified hypotheses | Introduction, final paragraph |
| Methods | 4 | Present key elements of study design early in the paper | Section 2.1 |
|  | 5 | Describe the setting, locations, and relevant dates, including periods of recruitment, exposure, follow-up, and data collection | Section 2.1 (first access January 2025 to April 2026) |
|  | 6a | Give the eligibility criteria, and the sources and methods of selection of participants | Section 2.2 and Figure 1 |
|  | 7 | Clearly define all outcomes, exposures, predictors, potential confounders, and effect modifiers | Sections 2.3 and 2.4 |
|  | 8 | For each variable of interest, give sources of data and details of methods of assessment; describe comparability of assessment methods if there is more than one group | Section 2.3; all instruments administered in routine care with the same protocol |
|  | 9 | Describe any efforts to address potential sources of bias | Section 2.2 (comparison of patients with and without the outcome measure); Section 2.4 (prespecified covariates, sensitivity analyses, multiple imputation) |
|  | 10 | Explain how the study size was arrived at | Sections 2.1 and 2.2: all patients with a first access in the study period; no a priori sample size calculation, as stated in Section 4.1 |
|  | 11 | Explain how quantitative variables were handled in the analyses, and which groupings were chosen and why | Section 2.3 (categories of internet use) and Section 2.4 |
|  | 12a | Describe all statistical methods, including those used to control for confounding | Section 2.4 |
|  | 12b | Describe any methods used to examine subgroups and interactions | Section 2.4 (comparisons across categories of internet use); no interaction analyses were performed |
|  | 12c | Explain how missing data were addressed | Section 2.4 (complete cases and extent of missingness; multiple imputation) |
|  | 12d | If applicable, describe analytical methods taking account of sampling strategy | Not applicable: all eligible patients in the period were included |
|  | 12e | Describe any sensitivity analyses | Section 2.4; results in Section 3.4 and Supplementary Table 1 |
| Results | 13a | Report numbers of individuals at each stage of study | Section 2.2 and Figure 1 |
|  | 13b | Give reasons for non-participation at each stage | Section 2.2 and Figure 1 |
|  | 13c | Consider use of a flow diagram | Figure 1 |
|  | 14a | Give characteristics of study participants and information on exposures and potential confounders | Section 3.1 and Table 1 |
|  | 14b | Indicate number of participants with missing data for each variable of interest | Table 1, column 'Valid n'; Section 2.4 |
|  | 15 | Report numbers of outcome events or summary measures | Section 3.1 and Table 1 |
|  | 16a | Give unadjusted estimates and, if applicable, confounder-adjusted estimates and their precision; make clear which confounders were adjusted for and why they were included | Sections 3.2 and 3.3 (unadjusted); Section 3.4 and Table 4 (adjusted, with 95% confidence intervals); covariate choice explained in Section 2.4 |
|  | 16b | Report category boundaries when continuous variables were categorized | Section 2.3 and Table 3 |
|  | 16c | If relevant, consider translating estimates of relative risk into absolute risk for a meaningful time period | Not applicable to a cross-sectional design with a continuous outcome |
|  | 17 | Report other analyses done | Section 3.4 (sensitivity analyses) and Section 3.5 (mediation); Supplementary Tables 1 to 3 |
| Discussion | 18 | Summarise key results with reference to study objectives | Discussion, first paragraph |
|  | 19 | Discuss limitations of the study, taking into account sources of potential bias or imprecision | Section 4.1 |
|  | 20 | Give a cautious overall interpretation of results considering objectives, limitations, multiplicity of analyses, results from similar studies, and other relevant evidence | Discussion; Section 4.2 |
|  | 21 | Discuss the generalisability (external validity) of the study results | Section 4.1 (single specialist service, age range, single recruitment window) |
| Other information | 22 | Give the source of funding and the role of the funders | Funding statement |

**RECORD Statement: checklist of items extending the STROBE statement, for studies conducted using observational routinely-collected health data**

| **Item** | **RECORD recommendation** | **Reported in** |
| --- | --- | --- |
| 1.1 | The type of data used should be specified in the title or abstract. When possible, the name of the databases used should be included | Abstract and Section 2.1: clinical data routinely collected at the Adult ADHD outpatient service |
| 1.2 | If applicable, the geographic region and timeframe within which the study took place should be reported in the title or abstract | Abstract (Milan, Italy); Section 2.1 (first access January 2025 to April 2026) |
| 1.3 | If linkage between databases was conducted for the study, this should be clearly stated in the title or abstract | Not applicable: no linkage between databases was performed |
| 2.1 | Explain the background, rationale and objectives with reference to the use of routinely collected data | Introduction, final paragraph; Section 2.1 |
| 3.1 | Provide the rationale for the use of the data source, and the population selected | Section 2.1 |
| 4.1 | The methods of study population selection should be reported, including the codes or algorithms used to identify patients | Section 2.2 and Figure 1; selection was based on the presence of a first access in the period and of a confirmed diagnosis at the structured interview, and not on administrative codes or algorithms |
| 6.1 | The methods of how the variables were extracted from the databases should be reported, together with a complete list of codes and algorithms used | Section 2.3; all variables were extracted directly from the clinical record fields, with no coding algorithm |
| 6.2 | Any validation studies of the codes or algorithms used should be referenced | Not applicable: no codes or algorithms were used; the psychometric instruments and their validation studies are referenced in Section 2.3 |
| 6.3 | If validation was conducted for this study and not published elsewhere, detailed methods and results should be provided | Not applicable |
| 7.1 | A complete list of codes and algorithms used to classify exposures, outcomes and confounders should be provided | Not applicable; the definition of every variable is given in Section 2.3 |
| 12.1 | Authors should describe the extent to which the investigators had access to the database population used to create the study population | Section 2.1: the investigators are the clinicians of the service and had access to the full clinical database for the period |
| 12.2 | Authors should provide information on the data cleaning methods used | Section 2.4 |
| 12.3 | State whether the study included person-level, institutional-level, or other data linkage across two or more databases | Not applicable: person-level data from a single service database, without linkage |
| 13.1 | Describe the extent to which the investigators had access to the database population used to create the study population, and report the number of individuals in the database population and in the study population | Section 2.2 and Figure 1 |
| 19.1 | Discuss the implications of using data that were not created or collected to answer the specific research question, including the misclassification bias, unmeasured confounding, missing data, and changing eligibility over time | Section 4.1 |
| 22.1 | Authors should provide information on how to access any supplemental information such as the study protocol, raw data, or programming code | Data availability statement and Supplementary Material |
