# Supplementary material for: Impulsivity is the principal correlate of problematic internet use in young adults with attention-deficit/hyperactivity disorder: a cross-sectional clinical study
Source: Front Psychiatry. 2026 Oct 28;17:1974570. doi: 10.3389/fpsyt.2026.1974570 (PMC13752202; doi:10.3389/fpsyt.2026.1974570)
Supplement: Supplementary file 2 [file Table2.docx]

**Supplementary Material**

**Supplementary Table 1. Sensitivity analyses for the association between impulsivity, emotional dysregulation and the Internet Addiction Test total score.**

| **Model** | **n** | **BIS-11 b (SE)** | **p** | **DERS b (SE)** | **p** | **Notes** |
| --- | --- | --- | --- | --- | --- | --- |
| S1. Main model (OLS) | 191 | 0.317 (0.071) | <0.001 | 0.091 (0.046) | 0.052 | adjusted R2 = 0.134 |
| S2. Main model, HC3 robust standard errors | 191 | 0.317 (0.067) | <0.001 | 0.091 (0.049) | 0.066 | same point estimates as S1 |
| S3. Adding interview-derived adult symptom counts | 191 | 0.326 (0.071) | <0.001 | 0.088 (0.047) | 0.060 | inattention p = 0.260; hyperactivity/impulsivity p = 0.378 |
| S4. Adding ASRS and WURS | 177 | 0.317 (0.072) | <0.001 | 0.113 (0.051) | 0.030 | ASRS p = 0.587; WURS p = 0.164 |
| S5. Adding ongoing psychotropic treatment | 190 | 0.304 (0.072) | <0.001 | 0.080 (0.047) | 0.089 | no psychotropic class significant |
| S6. Adding depressive and state anxiety symptoms | 173 | 0.333 (0.073) | <0.001 | 0.084 (0.052) | 0.109 | Zung p = 0.180; STAI-Y1 p = 0.719 |
| S7. Severity and education as categorical variables | 191 | 0.313 (0.071) | <0.001 | 0.085 (0.047) | 0.073 | adjusted R2 = 0.129 |
| S8. Multiple imputation (20 imputations) | 214 | 0.306 (0.065) | <0.001 | 0.095 (0.044) | 0.029 | chained equations |
| S9. Ordinal logistic model on the three IAT categories | 194 | OR per 10 points 1.37 | 0.001 | OR per 10 points 1.25 | <0.001 | outcome categorized, reported for completeness |

All models include sex, age, educational level, ADHD severity, clinical presentation (combined versus non-combined), current substance use disorder and any lifetime psychiatric comorbidity, in addition to the variables listed. Coefficients are unstandardized unless otherwise stated. BIS-11, Barratt Impulsiveness Scale; DERS, Difficulties in Emotion Regulation Scale; HC3, heteroscedasticity-consistent covariance estimator; OR, odds ratio; SE, standard error.

**Supplementary Table 2. Comparison between patients included in and excluded from the main multivariable model.**

| **Variable** | **Included in the main model** | **Excluded** | **p** |
| --- | --- | --- | --- |
| Age, years | 26.27 (n = 191) | 24.52 (n = 23) | 0.041 |
| IAT total | 52.62 (n = 191) | 55.74 (n = 23) | 0.319 |
| BIS-11 total | 81.21 (n = 191) | 87.30 (n = 20) | 0.114 |
| DERS total | 113.91 (n = 191) | 119.57 (n = 7) | 0.589 |
| ADHD severity (ordinal) | 2.16 (n = 191) | 2.13 (n = 15) | 0.768 |
| Female sex (proportion) | 0.43 (n = 191) | 0.32 (n = 22) | 0.319 |

Values are means unless otherwise stated; comparisons were performed with the Mann-Whitney U test. Patients were excluded from the main model only because of missing values on one or more covariates.

**Supplementary Table 3. Association between interview-derived symptom counts and the Internet Addiction Test total score.**

| **Measure** | **n** | **rho with IAT total** | **p** | **p (Holm)** |
| --- | --- | --- | --- | --- |
| Adult inattention (interview) | 205 | 0.012 | 0.866 | 1.000 |
| Adult hyperactivity/impulsivity (interview) | 205 | 0.119 | 0.088 | 0.353 |
| Childhood inattention (interview) | 206 | 0.004 | 0.951 | 1.000 |
| Childhood hyperactivity/impulsivity (interview) | 206 | 0.080 | 0.255 | 0.766 |

Spearman rank correlations. p (Holm) refers to adjustment with the Holm-Bonferroni procedure within this prespecified family of four tests. Symptom counts range from 0 to 9 criteria met.
